# SIRT3, a Mitochondrial NAD^+^-Dependent Deacetylase, Is Involved in the Regulation of Myoblast Differentiation

**DOI:** 10.1371/journal.pone.0114388

**Published:** 2014-12-09

**Authors:** Waed Abdel Khalek, Fabienne Cortade, Vincent Ollendorff, Laure Lapasset, Lionel Tintignac, Béatrice Chabi, Chantal Wrutniak-Cabello

**Affiliations:** 1 INRA, UMR866 Dynamique Musculaire et Métabolisme, F-34060 Montpellier - Université Montpellier 1, F-34000 Montpellier - Université Montpellier 2, Montpellier, France; 2 IGMM, Institut de Génétique Moléculaire de Montpellier, CNRS-UMR5535, Montpellier France - Université Montpellier 1, F-34000 Montpellier - Université Montpellier 2, Montpellier, France; 3 Biozentrum, University of Basel, Basel, Switzerland; Boston University School of Medicine, United States of America

## Abstract

Sirtuin 3 (SIRT3), one of the seven mammalian sirtuins, is a mitochondrial NAD^+^-dependent deacetylase known to control key metabolic pathways. SIRT3 deacetylases and activates a large number of mitochondrial enzymes involved in the respiratory chain, in ATP production, and in both the citric acid and urea cycles. We have previously shown that the regulation of myoblast differentiation is tightly linked to mitochondrial activity. Since SIRT3 modulates mitochondrial activity, we decide to address its role during myoblast differentiation. For this purpose, we first investigated the expression of endogenous SIRT3 during C2C12 myoblast differentiation. We further studied the impact of SIRT3 silencing on both the myogenic potential and the mitochondrial activity of C2C12 cells. We showed that SIRT3 protein expression peaked at the onset of myoblast differentiation. The inhibition of SIRT3 expression mediated by the stable integration of SIRT3 short inhibitory RNA (SIRT3shRNA) in C2C12 myoblasts, resulted in: 1) abrogation of terminal differentiation - as evidenced by a marked decrease in the myoblast fusion index and a significant reduction of Myogenin, MyoD, Sirtuin 1 and Troponin T protein expression - restored upon MyoD overexpression; 2) a decrease in peroxisome proliferator-activated receptor gamma coactivator 1-alpha (PGC-1α) and citrate synthase protein expression reflecting an alteration of mitochondrial density; and 3) an increased production of reactive oxygen species (ROS) mirrored by the decreased activity of manganese superoxide dismutase (MnSOD). Altogether our data demonstrate that SIRT3 mainly regulates myoblast differentiation via its influence on mitochondrial activity.

## Introduction

Skeletal muscle tissue is characterized by a high plasticity allowing tremendous metabolic adaptation in response to different physiological conditions. This flexibility occurs in parallel to changes in mitochondrial activity [1]. Recent studies have shown that mitochondria, besides their role in fuel metabolism, significantly influence muscle development, through the regulation of myoblast proliferation and differentiation, and the acquisition of contractile and metabolic features of muscle fibers [2], [3], [4], [5]. Indeed, mitochondrial activity controls myoblast differentiation via the regulation of c-Myc, Myogenin and Calcineurin expression. The same molecular targets are involved in the inhibitory effect of chloramphenicol, an inhibitor of mitochondrial protein synthesis, on myogenic differentiation. Conversely, upregulation of mitochondrial activity upon overexpression of the mitochondrial triiodothyronine receptor (p43) stimulates terminal differentiation [2], [4], [5].

Among the metabolic regulators, the sirtuin family, composed of seven NAD^+^ -dependent lysine deacetylases (NAD^+^: Nicotinamide Adenine Dinucleotide) is a group of metabolic sensors for cellular NAD^+^/NADH ratio. These proteins differ in tissue specificity, subcellular localization, enzymatic properties and targets [6]. Sirtuin1 (SIRT1), the most studied sirtuin, localizes to the nucleus where it deacetylates histones, transcription factors and their co-regulators. In muscle cells, SIRT1 interaction with MyoD and its co-activator P300/CBP-associated factor (PCAF) inhibits its function and prevents muscle differentiation [7]. Moreover, Fulco et al. reported that SIRT1 depletion, mediated by RNA interference, induces muscle cell differentiation in a non-permissive micro environment (AICAR insensitive) [8].

Three sirtuins are localized in mitochondria: SIRT3, SIRT4 and SIRT5, and participate in the regulation of ATP production, metabolism and cell signaling [9]. SIRT3 is considered as the major mitochondrial deacetylase since its depletion leads to mitochondrial protein hyperacetylation, an event not occurring after SIRT4 or SIRT5 inhibition [10]. In agreement with these observations, recent studies have established that, in addition to a weak deacetylase activity, SIRT4 and SIRT5 have other functions; SIRT4 exerts an inhibitory ADP-ribosyl-transferase activity towards the glutamate dehydrogenase (GDH) [11] and SIRT5 was reported to exert a desuccinylase/demalonylase activity [12].

The first identified SIRT3 target was the mitochondrial protein acetyl-coenzyme A (acetyl CoA) synthase 2 (AceCS2) which requires deacetylation in order to convert acetate to acetyl CoA in the presence of ATP [13], [14]. Similar positive effects are as well described upon SIRT3 dependent deacetylation of the glutamate dehydrogenase (GDH), an enzyme required for urea synthesis, and the long-chain acyl CoA dehydrogenase (LCAD), a central enzyme in the fatty acid oxidation pathway [15]. Moreover, SIRT3 modulates the production of cellular ROS *via* deacetylation of antioxidant enzymes such as superoxide dismutase 2 (SOD2, MnSOD) [16], [17]. SIRT3 also controls ATP levels by modulating the activity of the respiratory chain complexes I and II upon binding to NDUFA9 [18] and SdhA [19] subunits respectively.

Consequently, it becomes increasingly clear that reversible lysine acetylation is a major post-translational modification of the mitochondrial proteome central for the maintenance of their proper function and for the adaptation of mitochondrial activity. In turn, our group previously described the involvement of mitochondrial activity in the regulation of myoblast differentiation and myogenic factor expression and/or activity. Since SIRT3 does modulate mitochondrial activity, we have investigated here its influence on myoblast differentiation.

## Materials and Methods

### Cell culture

Mouse myoblasts of the C2C12 cell line (ATCC) were grown in Dulbecco's modified Eagle's medium (DMEM) containing 4.5 g/l glucose, 0.584 g/l L-glutamine, and 3.7 g/l sodium bicarbonate and supplemented by gentamycin (50 µg/ml), amphotericin (50 µg/ml), and Fetal Calf Serum (FCS, 10%). Terminal differentiation was induced when cells reached 80% of confluence, by replacing FCS by horse serum (2%). The differentiation medium was changed every two days.

### SIRT3shRNA

pTER-shSIRT3 expression vectors were generated with siRNAs against murine SIRT3 (Santa Cruz *sc-61556A and sc-61556B*) inserted into pTER vector at *Bgl*II and *Hind*III sites, using the following two oligonucleotides pairs [20]:

5′gatcCC*AAGTAGTGAGTGACATTGG*TTAGTGAAGCCACAGATGTAA*CCAATGTCACTCACTACTT*TTTTTGGAAA-3′and

5′agctTTTCCAAAAA*AAGTAGTGAGTGACATTGG*TTACATCTGTGGCTTCACTAA*CCAATGTCACTCACTACTT*GG-3′ for clone series 1;

5′gatcCC*ATATGGAGTAGGAACCTTG*TTAGTGAAGCCACAGATGTAA*CAAGGTTCCTACTCCATAT*TTTTTGGAAA-3′ and

5′agctTTTCCAAAAA*ATATGGAGTAGGAACCTTG*TTACATCTGTGGCTTCACTAA*CAAGGTTCCTACTCCATAT*GG -3′ for clone series 2.

To obtain pTER-shLuc control vector expressing siRNA against firefly luciferase, the following oligonucleotide pair was inserted into pTER:

5′GATCCCCGTACGCGGAATACTTCGATTCAAGAGATCGAAGTATTCCGCGTACGTTTTTGGAAA-3′ and

5′AGCTTTTCCAAAAACGTACGCGGAATACTTCGATCTCTTGAATCGAAGTATTCCGCGTACGGG-3′. All construct sequences were confirmed by sequences analyses.

These vectors were transfected into C2C12 cells using the JetPEI transfection reagent (Polyplus transfection) according to the manufacturer's instructions. Clones stably expressing SIRT3 or Luc shRNA were selected 48h after transfection by addition of 0.8 mg/ml Zeocin and positive clones were further maintained in 0.4 mg/ml Zeocin.

### Retrovirus Production

Retroviral vector pBabe-MyoD was generated using the pEMSV-MyoD expression vector [21] and the retroviral backbone pBabe-puro (Cell Biolabs). The 1.7 kb murine MyoD fragment was cut with EcoRI and then inserted into pBabe-puro at EcoRI sites.

Retroviral constructs were transiently transfected using the JetPEI transfection reagent into Plat-E packaging cells to produce non-replicating retrovirus and the supernatant was harvested.

### Retroviral infection

C2C12 cells were seeded at 8×10^3^ cells/cm^2^ and grown in proliferative medium. When cells reached 50% of density, the medium was replaced with a 2∶1 dilution of Plat-E retroviral supernatant with 8 µg/ml polybrene and incubated at 37°C. The next day, the cells were rinsed and placed in fresh medium, and allowed to reach confluence. For MyoD rescue on SIRT3shRNA clones, myogenic cells were analysed at confluence, and following differentiation for one to three days.

### Cytoimmunofluorescence and fusion index

Myoblast differentiation was assessed by morphological changes and accumulation of muscle-specific markers. After fixation with 3% paraformaldehyde for 30 min, cells were permeabilized 10 min in 0.25% Triton X-100 and saturated in PBS–FCS (5%). Revelation was performed using an anti-Troponin T or α-Tubulin antibody (Sigma, 1∶100 and 1∶500 respectively) and detected with a photoprobe 488 secondary antibody (anti-Mouse IgG). Nuclei were stained with Hoechst 33258 (1 µg/ml). All images were digitalized using the Kappa image base analysis system.

To calculate the fusion index, the number of nuclei incorporated into myotubes (>2 nuclei) was assessed and the ratio of this number to the total number of nuclei was determined. Nuclei were counted on 10 images/dish using Image J software.

### Immunoblotting

Cells were lysed in Tris-NP40 buffer (50 mM Tris, pH 8.0, 150 mM NaCl, 1% Nonidet P-40) supplemented with a protease inhibitor cocktail (Roche Diagnostics). Total protein concentration was determined using the Bio-Rad protein assay.

Fifty µg of proteins were run on SDS-PAGE mini-gels at the appropriate concentration of acrylamide and transferred onto nitrocellulose membrane. Membranes were blocked (1 h at room temperature) with a 5% skim milk in 1×TBST (Tris-buffered saline Tween-20: 20 mM Tris-HCl, pH 7.6, 137 mM NaCl, and 0.2% Tween-20) solution and probed with an antibody raised against SIRT3, SIRT1 (Cell Signaling, 1∶1000), Myogenin, MyoD (Santa Cruz Biotechnology, 1∶50), voltage-dependent anion-selective channel (VDAC) (Abcam 1∶3000), Citrate synthase (Genetex, 1∶1000), PGC-1α (Calbiochem,1∶500) and Tubulin (Sigma, 1∶10000) overnight at 4°C. After washes with TBST, blots were incubated at room temperature (1 h) with the appropriate secondary antibody coupled to horseradish peroxidase and washed again. Antibody-bound protein was revealed using the ECL reagent (Thermo Scientific). Films were scanned and analyzed using Image J software. All blots were corrected for loading using Tubulin expression.

### Real-time PCR analysis of mRNA levels

Total RNA was extracted from cells using the Trizol reagent (Invitrogen Life Technologies) as recommended by the manufacturer. RNA concentration was determined spectrophotometrically (λ = 260 nm) and integrity of mRNA was analyzed on agarose gel.

Reverse transcription reaction was performed with 1 µg of total RNA using SuperScript First-strand synthesis system, with 50 units of Superscript II reverse transcriptase, random hexamers, and Oligo (dT) primers (Invitrogen Life Technologies) according to the manufacturer's instructions. Reverse transcription was performed simultaneously for all samples.

mRNA gene expression was determined by Real-time Quantitative Polymerase Chain Reaction (RT-qPCR). RT-qPCR analyses were performed in a MiniOpticon detection system (Biorad, Hercules, CA, USA) with 7.5 µl of IQ^TM^ SYBR Green Supermix (Biorad, Hercules, CA, USA), 200 nM of both forward (Sirt3-For: 5′-CTGACTTCGCTTTGGCAGAT-3′) and Reverse (Sirt3-Rev: 5′-GTCCACCAGCCTTTCCACAC-3′) primers, 2 µl of cDNA template, and water to a final volume of 15 µl. Primers were designed using Universal Probe Library Assay Design Center (Roche Applied System) and RT Primer Data Base.

PCR was performed in duplicate using the following cycle parameters: 30 s at 98°C, followed by 40 cycles of 1 s at 92°C and 15 s at 60°C. Melting point dissociation curves were performed between 65°C and 95°C (temperature transition of 0.5°C) to confirm that only a single product was amplified. To ensure quality of the measurements, each PCR experiment for each gene included a negative control (sample replaced by RNase free water). Results were expressed using the comparative cycle threshold (Ct) method (CFX Manager, Biorad): the (ΔΔCt values were calculated in every sample for each gene of interest with TBP (TBP-For: 5′ -TGTGCACAGGAGCCAAGA-3′, TBP-Rev: 5′-CCCCACCATGTTCTGGAT-3′) and ARP (ARP-For: 5′-ACTGGTCTAGGACCCGAGAAG-3′, ARP-Rev: 5′-TCCCACCTTGTCTCCAGTCT-3′) as the reference genes. All results are expressed relative to shCTL cells in proliferative state and presented as means ± SD.

### Mitochondrial isolation

Mitochondria were isolated as previously described by Frezza et al. [22]. Briefly, cells were pelleted by centrifugation for 10 min at 600 g and resuspended in ice-cold isolation buffer (IBc; 10 mM Tris–MOPS, 1 mM EGTA/Tris, and 200 mM sucrose, pH 7.4). Cells were homogenized with a motor-driven glass–Teflon potter at 1,600 rpm for 5 min.

Nuclei and unbroken cells were removed by centrifugation for 10 min at 600 g at 4°C and mitochondria were pelleted from the supernatant by further centrifugation for 10 min at 7000 g at 4°C. Mitochondria were resuspended in IBc, and protein content was determined using the Bradford assay.

### Enzyme activity assays

The maximal enzymatic activity of mitochondrial respiratory chain complexes and Citrate synthase (CS) were measured in SIRT3shRNA and LucshRNA clones at confluence and on the third day of differentiation. Complex II (CII), Cytochrome c oxidase (COX) activities were measured spectrophotometrically according to Rustin et al. [23] and Wharton et al. [24]; CS activity was measured according to Srere [25]. MnSOD activity was measured on isolated mitochondria according to Marklund [26].

### Respiration

Cell oxygen consumption was measured using the high-resolution Oxygraph-2k (OROBOROS Instruments, Innsbruck, Austria). Cells were incubated in two sealed thermostated chambers (37°C) containing 2 ml of MIRO5 respiration medium [0.5 mM EGTA, 3 mM MgCl_2_·6H_2_O, 65 mM KCl, 20 mM taurine, 10 mM KH_2_PO_4_, 20 mM HEPES, 110 mM sucrose, and 1 g/l BSA, pH 7.1] [27]. Basal respiration was evaluated after closing the chambers. Maximal respiration was determined after blocking ATP-synthase activity by oligomycin (2.5 µM) and adding successive amounts of 0.2 µM CCCP to achieve maximal oxygen consumption. Data acquisition and analysis were performed using Oxygraph-2k-DatLab software version 4.3.2.7 (OROBOROS Instruments).

### Measurement of intracellular ROS

ROS accumulation was measured using the 2′, 7′-dihydrodichlorofluorescein-diacetate (H_2_DCF-DA) probe (Invitrogen). SIRT3shRNA or LucshRNA control cells grown on 24-well plate, were washed with Locke buffer (140 mM NaCl; 5 mM KCl; 1.2 mM MgCl_2_; 1.8 mM CaCl_2_; 10 mM glucose; 10 mM Hepes, 1 M Tris-HCl pH 7.5) and then incubated with 10 µM H_2_DCF-DA probe in Locke buffer for 20 minutes at 37°C. After a quick wash, fluorescence measurement (λex: 485/λem: 530 nm) was performed using Synergy2 microplate reader (Biotek France, Colmar, France) for 1 h. To account for the cell number in each cellular state, H2DCF-DA fluorescence was normalized using DNA content as previously described Laguerre et al. [28].

### Statistical analysis

Data are represented as mean ± SD from at least three independent experiments. A Student's t-test was used to determine the effects of cell state on protein expression and of cell clones on fusion index. A two-way ANOVA followed by Bonferroni's pairwise multiple-comparison test was used to determine the effects of cell state and cell clones on protein expression, mitochondrial respiration, mitochondrial complex enzyme activities and ROS production. For all tests, statistical significance was set for *P* value*<*0.05. The data were analyzed using the statistical package GraphPad Prism.

## Results

### SIRT3 expression during C2C12 differentiation

**Figure 1:**
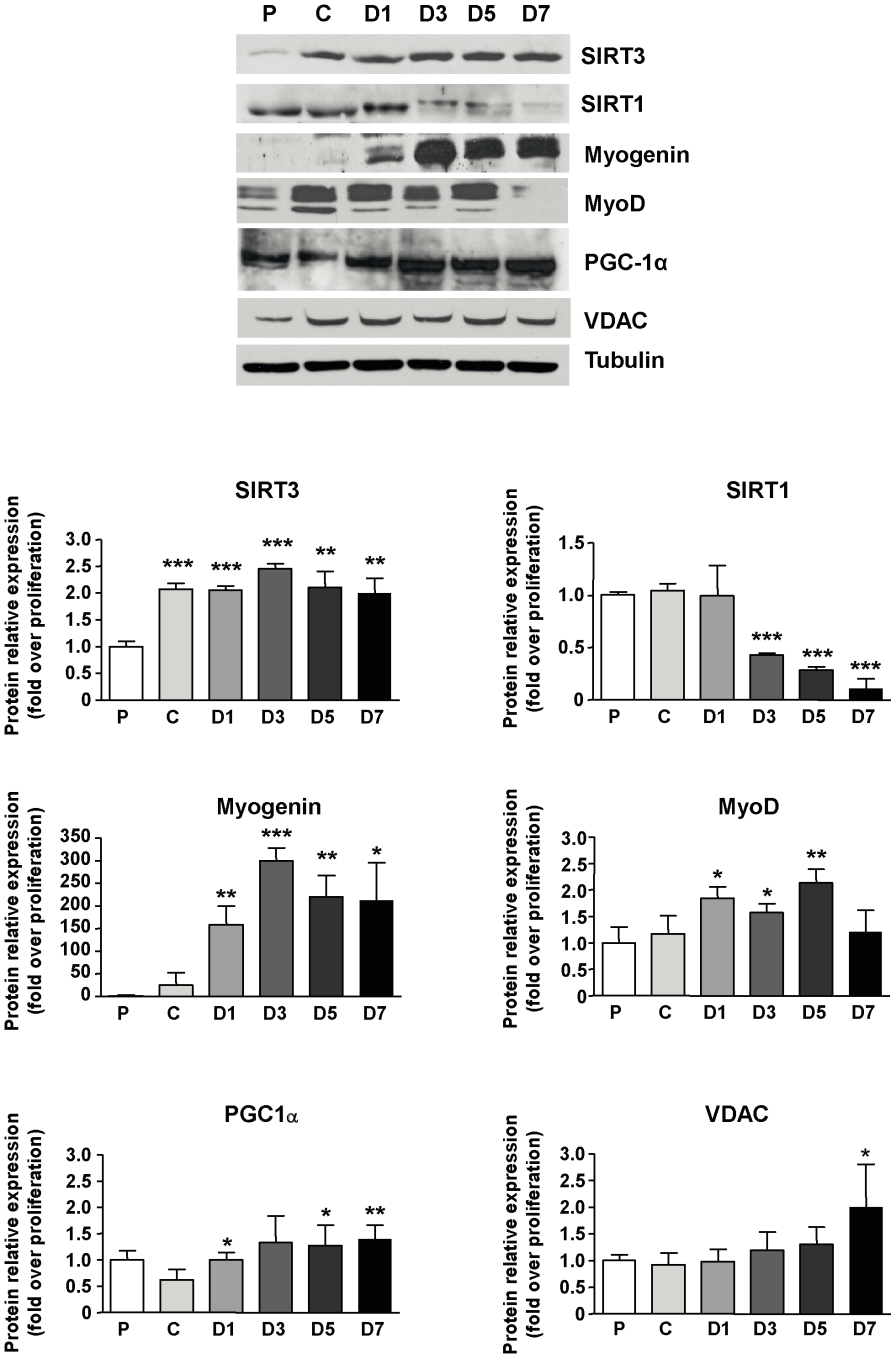
Changes in the expression of the SIRT3 protein during C2C12 myoblast differentiation. Western-blot analyses of SIRT3, SIRT1, Myogenin, MyoD, PGC-1α and VDAC protein expressions during proliferation (P), at cell confluence (C) and after 1, 3, 5, and 7 days of differentiation (D1, D3, D5, and D7 respectively). Representative blots are shown. Quantification was performed with Image J software and normalized relatively to Tubulin protein levels. Results are expressed as the mean ± SD of three separate experiments. *P<0.05, **P<0.01 and ***P<0.001 *vs.* proliferating myoblasts for SIRT3, SIRT1, Myogenin, MyoD and *P<0.05, **P<0.01 *vs.* confluent myoblasts for PGC-1α and VDAC.

To determine the expression profile of sirtuins during C2C12 myogenic differentiation, expression levels of SIRT1 and SIRT3 protein were quantified at different time points of C2C12 cell differentiation determined by the western blot detection of myogenic marker expression (Fig. 1). SIRT3 expression, hardly detectable in proliferating myoblasts, increased sharply at cell confluence (P<0.001) and stayed elevated throughout differentiation. In contrast, SIRT1 protein levels, high in proliferating myoblasts, declined when cells undergo terminal differentiation with a marked decrease at differentiation day 3, down to the lowest expression level detectable at differentiation day 7 (P<0.001).

As expected, Myogenin expression occurred at the onset of terminal differentiation and reached a maximal value on day 3 of differentiation (P<0.001). MyoD protein expression increased 24 h after the induction of differentiation and remained higher than proliferating myoblasts until day 5 of differentiation.

PGC-1α protein level significantly increased on the first day of differentiation and remained elevated during terminal differentiation (P<0.01, day 7 vs. proliferation). VDAC protein level progressively increased during differentiation, up to 2-fold at day 7 of differentiation (P<0.05 vs. proliferation).

### shRNA knockdown of endogenous SIRT3 alters myogenic differentiation

In order to investigate whether the early upregulation of SIRT3 expression reflects its functional involvement in myogenic differentiation, we silenced SIRT3 expression in C2C12 myoblast using specific shRNA (SIRT3shRNA).

**Figure 2:**
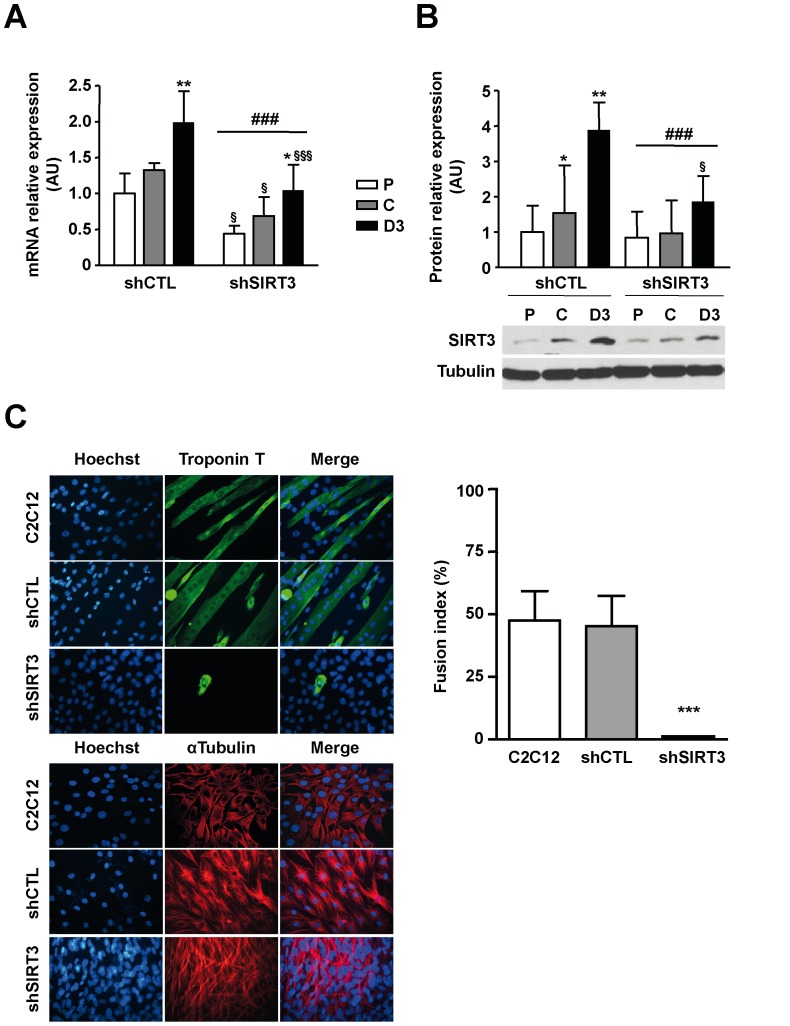
Depletion of SIRT3 impairs terminal myoblast differentiation. Endogenous SIRT3 expression in C2C12-LucshRNA (shCTL) and C2C12-SIRT3shRNA (shSIRT3) cells during proliferation (P), at cell confluence (C) and after 3 days of differentiation (D3). A) SIRT3 mRNA expression was monitored by real-time RT-PCR and normalized relatively to the reference genes ARP and TBP. Results are expressed as the mean ± SD of three independent experiments. B) Western blot analysis of SIRT3 protein expression. Quantification was performed with Image J software and normalized relatively to Tubulin protein level. Results are expressed as the mean ± SD of three separate experiments. ANOVA main effect: ### P<0.001 *vs.* shCTL cells. Post-hoc significance: *P<0.05, **P<0.01 and ***P<0.001 *vs.* proliferating myoblasts for each cell type. §P<0.05, §§P<0.01 and §§§ P<0.001 *vs.* shCTL cells at the same statstatee. C) Immunostaining of C2C12, C2C12-LucshRNA (shCTL) and C2C12-SIRT3shRNA (shSIRT3) cells with an anti-Troponin T (differentiated state) or anti α-tubulin (undifferentiated state) antibody and fusion index 3 days after the induction of differentiation. Nuclei were stained with Hoechst. Microphotographs of a typical experiment are shown (x400). Fusion index values are expressed as the mean ± SD of 10 images/dish using image J software. *** P<0.001 *vs.* shCTL cells.

Several myoblast clones displaying a moderate to strong decrease in SIRT3 mRNA levels were generated. The clone selected to conduct the experiments displayed a moderate inhibition of SIRT3 mRNA expression level (3 days of differentiation: −47% relative to control; P<0.001; Fig. 2A) similar to the downregulation observed at the protein level (P<0.001; Fig. 2B)

SIRT3 depletion resulted in the inhibition of C2C12 terminal differentiation as reflected by the dramatic decrease of myoblast fusion index recorded at day 3 of differentiation (shSIRT3: −100% relative to shCTL, P<0.001; Fig. 2C). Immunocytochemistry detection of the differentiation marker Troponin T and the cytoskeletal α-tubulin confirmed that terminal differentiation was strongly inhibited in shSIRT3 cells (Fig. 2C). A similar impairment of C2C12 differentiation was seen in other shSIRT3 clones (data not shown).

**Figure 3:**
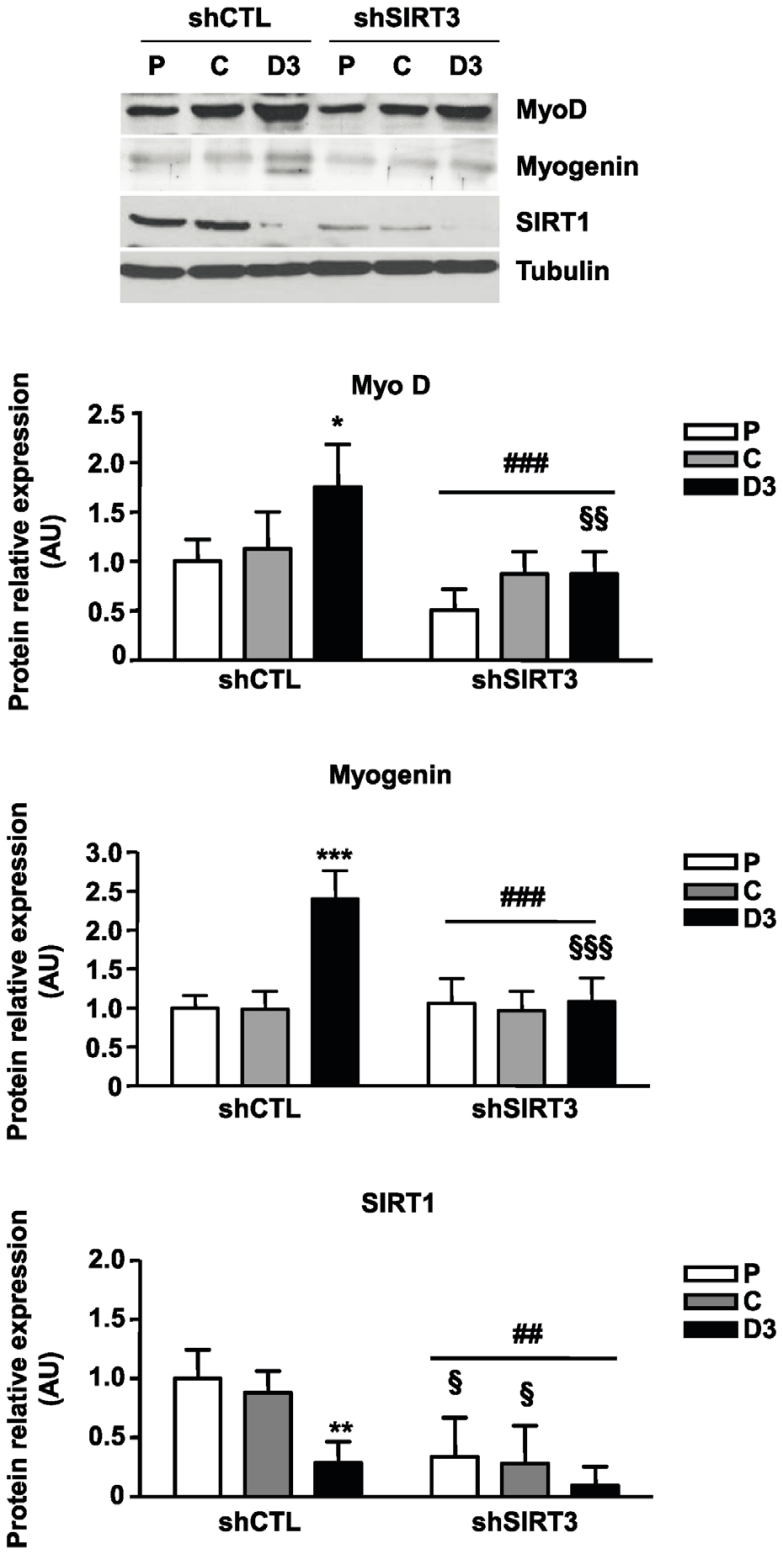
Depletion of SIRT3 impairs Myogenin, MyoD and SIRT1 expression. Western-blot analyses of Myogenin, MyoD, Sirt1 and Tubulin in C2C12-LucshRNA (shCTL) and C2C12-SIRT3shRNA (shSIRT3) cells at the indicated states (Proliferation, P; Cell Confluence, C and 3 days after the induction of differentiation D3). Results are normalized relatively to Tubulin protein levels and expressed as the mean ± SD of three separate experiments. ANOVA main effect: ## P<0.01, ### P<0.001 *vs.* shCTL cells. Post-hoc significance: *P<0.05, **P<0.01 and ***P<0.001 *vs.* proliferating myoblasts for each cell type. §P<0.05, §§P<0.01 and §§§ P<0.001 *vs.* shCTL cells at the same stage.

SIRT3 depletion was accompanied by a significant decrease in MyoD expression at day 3 of differentiation (−50%; P<0.01; Fig. 3) and fully abrogated the rise in Myogenin protein expression normally occurring at day 3 of differentiation in control C2C12 cells (−55%, P<0.001; Fig. 3).

Although generally considered as a negative regulator of myoblast differentiation, we observed that SIRT1 protein expression was considerably decreased in SIRT3 depleted cells (P: −65%, C: −69% and D3: −69% relatively to control cells, P<0.01, Fig. 3).

### MyoD overexpression restores differentiation of SIRT3shRNA myoblast

We showed that SIRT3 silencing in myoblasts resulted in the blockade of myogenic differentiation and myotube formation by inhibiting expression of MyoD and Myogenin, its downstream effector. We decided to test whether MyoD overexpression could overcome the pattern of differentiation seen in the SIRT3 depleted cells.

**Figure 4:**
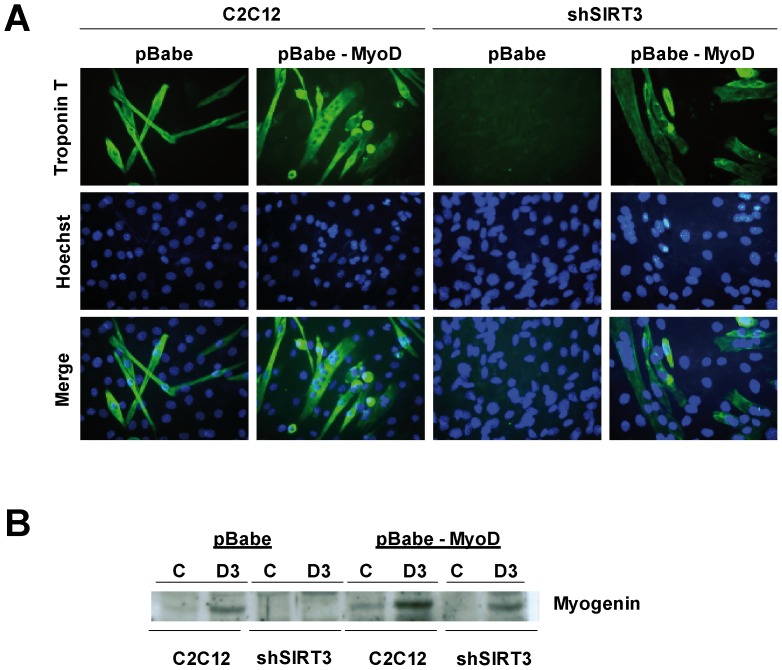
MyoD overexpression in shSIRT3 restores myoblasts differentiation. C2C12 and C2C12-SIRT3shRNA (shSIRT3) cells were infected with pBabe empty vector and pBabe-MyoD vector retroviral supernatants. A) Immunostaining with an anti-Troponin T antibody 3 days after the induction of differentiation. Nuclei were stained with Hoechst. Microphotographs of a typical experiment are shown (x400). B) Representative Western-blot of Myogenin at cell confluence (C) and after 3 days of differentiation (D3).

As expected, transient MyoD overexpression strongly stimulated C2C12 myoblast terminal differentiation (assessed by morphological criteria, Troponin T accumulation Fig. 4A) associated with an increase in Myogenin expression (Fig. 4B). Transient infection with MyoD in shSIRT3 myoblasts restored differentiation to levels found in normal C2C12 myoblasts, as shown by myotube formation, positive Troponin T immunostaining and increased Myogenin expression of the infected cells (Fig. 4).

### Influence of SIRT3 down-regulation on mitochondrial activity

To address the effect of SIRT3 depletion on mitochondrial activity and biogenesis, we measured several parameters such as respiratory ratio, enzymatic activities of the respiratory chain complexes involved in substrate oxidation, and ROS accumulation.

**Figure 5:**
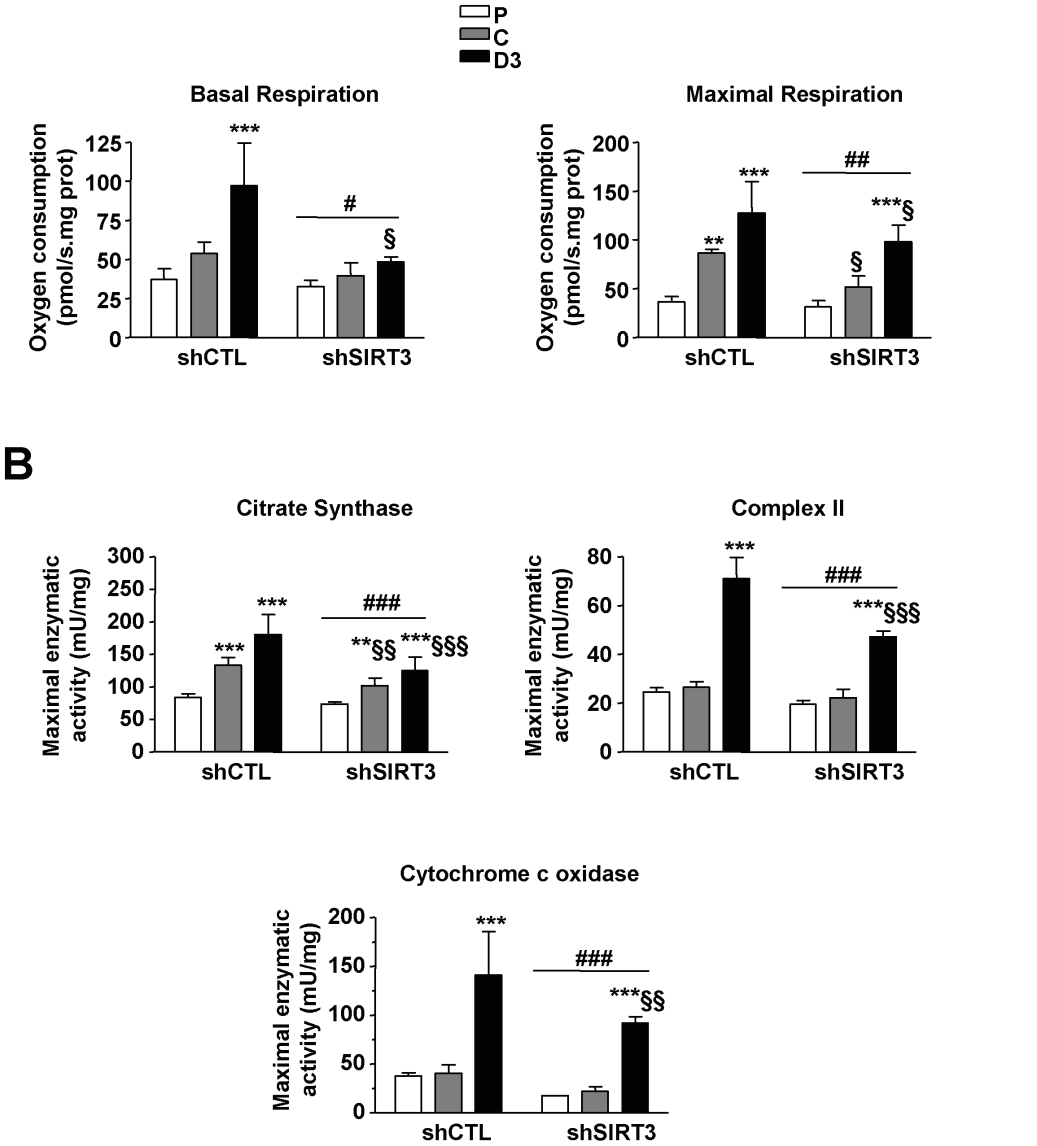
Influence of SIRT3 depletion on mitochondrial activity. A) Respiration rates in C2C12- LucshRNA (shCTL) and C2C12-SIRT3shRNA (shSIRT3) at cell proliferation (P), cell confluence (C) and 3 days of differentiation (D3). Values are normalized relatively to total protein levels. B) Citrate synthase, complex II and cytochrome c oxidase maximal activities in shCTL and shSIRT3 cells at cell proliferation (P), cell confluence (C) and 3 days of differentiation (D3). Results are expressed as mean ± SD from five independent experiments. ANOVA main effect: #P<0.05, ##P<0.01 and ###P<0.001*vs.* shCTL cells. Post-hoc significance: *P<0.05, **P<0.01 and ***P<0.001 *vs.* proliferating myoblasts for each cell type. §P<0.05, §§P<0.01 and §§§ P<0.001 *vs.* shCTL cells at the same state.

In control cells, the basal respiration rate significantly increased from the proliferation state to the third day of differentiation (Fig. 5A), (P *vs.* D3: +161.3%, P<0.001). Such changes were not observed in SIRT3 depleted myoblasts (P *vs.* D3: +48.5%, P = NS). Of note, both control (P *vs.* D3 +250%, P<0.001) and SIRT3 depleted cells (P *vs*. D3 +212.5%, P<0,001; Fig. 5A) increased their maximal respiration rate in response to CCCP treatment (from cell proliferation to day 3 of differentiation). However, basal and maximal O_2_ consumptions were lower during differentiation in SIRT3shRNA cells when compared to control cells (−49.8% and −30.0% respectively, P<0.05; Fig. 5A).

As expected, citrate synthase activity, succinate dehydrogenase (complex II) and cytochrome c oxidase (COX) activities, all significantly increased from proliferation to day 3 of differentiation in control cells (P *vs*. D3: +116%, +190% and +273% respectively, P<0.001; Fig. 5B), while a rise was also observed from proliferation to day 3 of differentiation in SIRT3 depleted cells (P *vs*. D3: +70%, +142% and +430%, respectively, P<0.001; Fig. 5B). However, these activities were significantly lower in SIRT3 depleted cells than in control cells at day 3 of differentiation (CS: −31%, Complex II: −33%, COX, −35% P<0.001; Fig. 5B).

**Figure 6:**
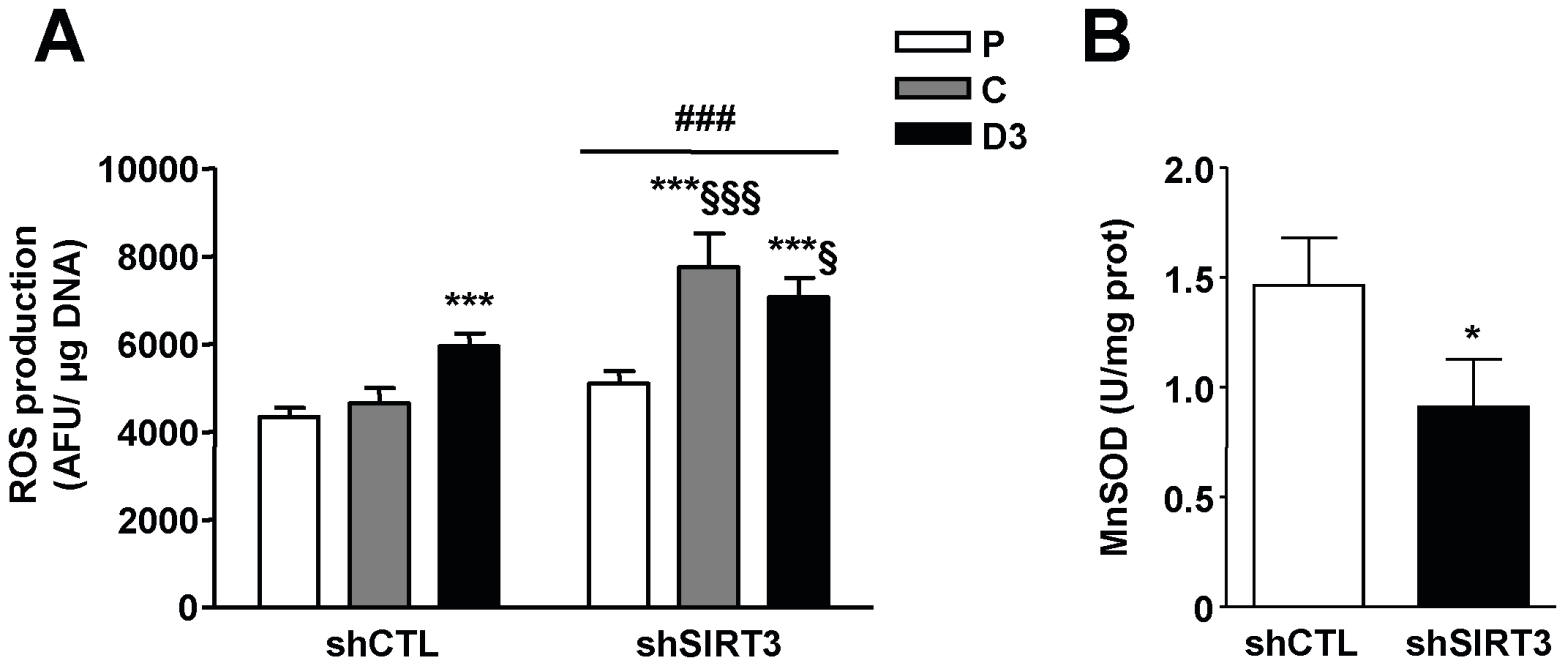
SIRT3 depletion induces an oxidative stress. A) Intracellular ROS accumulation in C2C12- LucshRNA (shCTL) and C2C12-SIRT3shRNA (shSIRT3) at cell proliferation (P), cell confluence (C) and 3 days of differentiation (D3). Values are normalized relatively to total DNA levels. ANOVA main effect: ### P<0.001 *vs.* shCTL cells. Post-hoc significance: ***P<0.001 *vs.* proliferating myoblasts for each cell type. §P<0.05, §§§ P<0.001 *vs.* shCTL cells at the same state. B) MnSOD maximal activity in shCTL and shSIRT3 cells cell confluence (C). Results are expressed as mean ± SD from five independent experiments. *P<0.05 *vs.* shCTL cells.

In controls cells, the amount of intracellular ROS levels was significantly increased at day 3 of differentiation (P *vs*. D3 +37%, P<0.001; Fig. 6A), while a rise was also observed in SIRT3 depleted cells from cell confluence (P *vs*. C: +22%, P<0.001; P *vs*. D3: +12%, P<0.001; Fig. 6A). SIRT3 depletion increased the amount of intracellular ROS levels compared to control cells (+47% in proliferating cells; +67% at cell confluence; and +20% at day 3 of differentiation, P<0.001; Fig. 6A). Moreover, MnSOD, a target of SIRT3, displayed a significantly decreased activity in SIRT3-depleted cells when compared to control cells (−40%, P<0.05; Fig. 6B).

**Figure 7:**
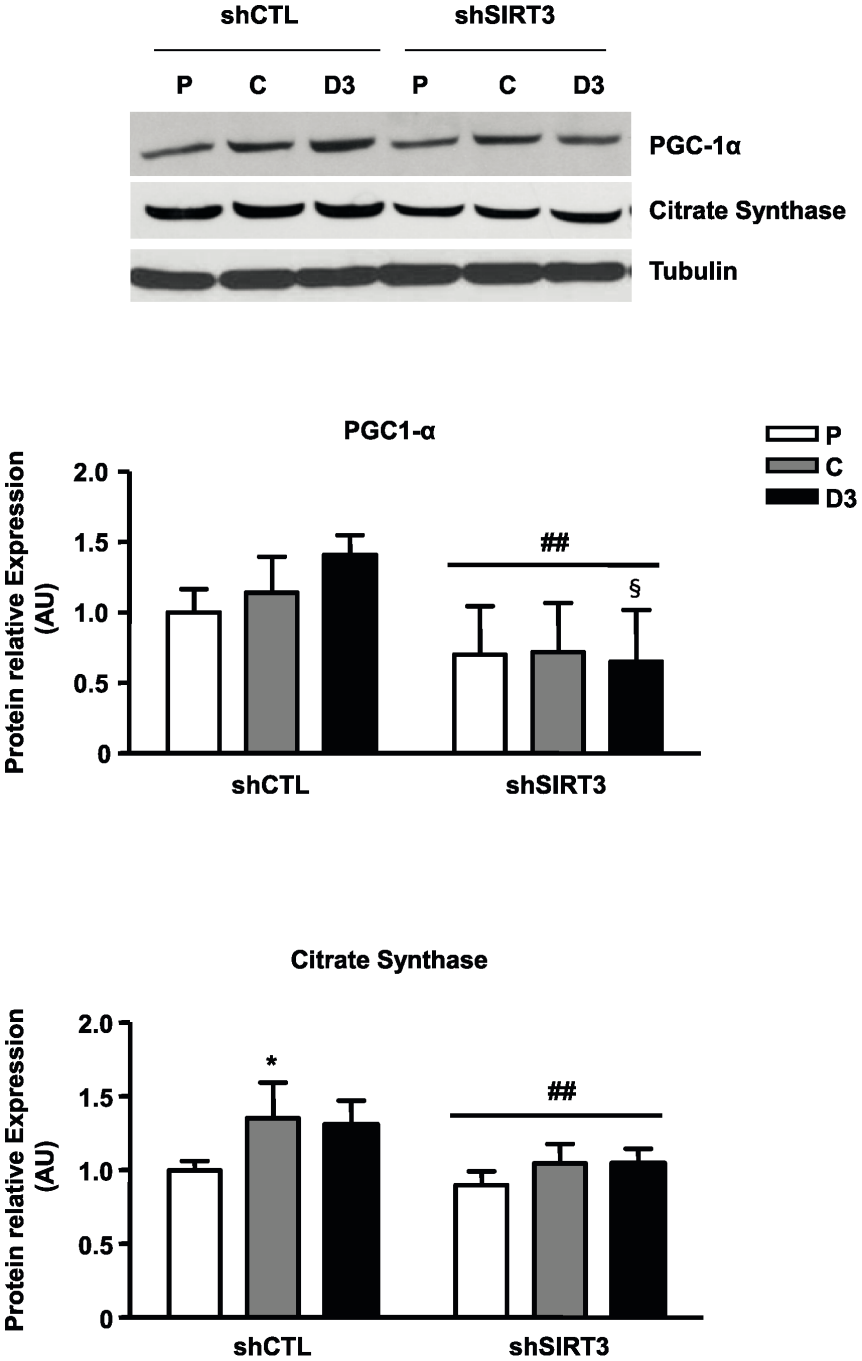
Influence of SIRT3 depletion on protein expression of mitochondrial content markers. At the indicated states (Proliferation, P; Cell Confluence, C and 3 days after the induction of differentiation, D3), 50 µg of total protein from C2C12-LucshRNA (shCTL) and C2C12- SIRT3shRNA (shSIRT3) were immunoblotted with antibodies raised against PGC-1α, CS or Tubulin as an internal control. Results are expressed as the mean ± SD of three separate experiments. Quantification was performed with Image J software and normalized relatively to Tubulin protein levels. ANOVA main effect: ## P<0.01 *vs.* shCTL cells. Post-hoc significance: *P<0.05 *vs.* proliferating myoblasts for each cell type. §P<0.05 *vs.* shCTL cells at the same state.

In order to test the influence of SIRT3 on mitochondrial biogenesis, we measured the expression of markers of the mitochondrial mass: PGC-1α, a major regulator of mitochondrial biogenesis and citrate synthase (Fig. 7). In shSIRT3 cells, PGC-1α and citrate synthase proteins level failed to increase during differentiation, with a significant decrease in PGC-1α protein level at day 3 of differentiation, when compared to control cells (−46%, P<0.05).

## Discussion

Development and tissue growth require complex cellular mechanisms to meet cellular energy needs for protein, DNA and phospholipid synthesis, and expression of target genes. Developmental processes require a tight coordination between the expression of genes involved in cell specification, proliferation, differentiation, apoptosis, and the genes that optimize fuel metabolism. Consequently, genes involved in both tissue elaboration and metabolism are of particular interest. Among these genes, members of the sirtuin deacetylase family, the metabolic sensors responsive to cellular NAD+/NADH ratio, regulate the activity of target genes considered as major differentiation effectors. In particular, it has been shown that SIRT1 nuclear deacetylase regulates the activity of PGC-1α [29] and MyoD [7].

Similarly, previous studies have established that changes in mitochondrial protein synthesis greatly influence myoblast differentiation [2], [4], [5]. Consequently, it would be expected that changes in mitochondrial proteins activity would also regulate these myogenic processes. Among mitochondrial sirtuins, the major mitochondrial deacetylase SIRT3 upregulates the activity of several proteins inside the organelle, leading to stimulation of mitochondrial activity [10]. Taken together, these results suggest a possible involvement of SIRT3 in the regulation of myoblast differentiation. However, the relationships between SIRT3 and myogenesis have not been studied yet.

Initially, we studied the pattern of SIRT3 expression during myoblast differentiation in parallel to that of major myogenic effectors, and mitochondrial biogenesis markers. As expected, increase in MyoD and Myogenin protein levels were in agreement with previous studies [30]. Myogenin expression was induced on the first day of differentiation whereas MyoD expression increased moderately during differentiation from D1 to D5. Similarly, as already shown [31], PGC-1α expression increased at the onset of differentiation and remained higher than that in confluent myoblasts. As PGC-1α is a master regulator of mitochondrial biogenesis [32], and in agreement with other studies [31], [33], [34], our results confirmed that myoblast differentiation is associated with a stimulation of mitochondriogenesis.

As reported by Fulco et al. [7], we observed that SIRT1 expression, elevated in proliferating myoblasts, sharply decreased during terminal differentiation. Because this sirtuin is considered to be a potent repressor of myoblast differentiation mainly through the inhibition of MyoD activity [7], alterations of SIRT1 expression modulates the progression of the myogenic process. Interestingly, SIRT3 expression displayed a bigger and longer lasting increase than myogenin at the very onset of differentiation, suggesting a possible involvement of SIRT3 on the myogenic process.

In agreement, SIRT3 depletion blocked myoblast differentiation; we could not detect polynucleated myotubes three days after the induction of differentiation (medium change), and Troponin T, an early marker of differentiation, was barely or not detected in these cells. This influence was associated with a significant decrease of Myogenin expression, a myogenic transcription factor requisite for terminal differentiation, which did not increase after the induction of differentiation as shown in control myoblasts. Similarly, MyoD protein expression was significantly lower in SIRT3 depleted cells when compared to control ones, and did not display a differentiation-induced rise. These data suggested that inhibition of MyoD expression induced by SIRT3 depletion could be responsible for the alteration of Myogenin expression, a direct MyoD target [35]. Interestingly, overexpression of MyoD in SIRT3-depleted myoblasts restored Myogenin expression and the fusogenic potential of these cells indicating that the activity of the myogenic factor is not affected in shSIRT3 myoblasts. Thus, SIRT3 depletion impaired myogenic differentiation through repression of MyoD expression, a master regulator of skeletal myogenesis. Our data suggested that silencing of SIRT3 might either interfere with a positive regulator of MyoD expression or stabilize a repressor of *MyoD* transcription.

Another striking result was the observation that SIRT3 depletion strongly inhibited SIRT1 expression. As endogenous SIRT1 protein levels decreased during differentiation, these changes did not result from the differentiation block. Instead SIRT3 may directly or indirectly regulate SIRT1 expression level, providing a fine tuning of myoblast differentiation through a regulatory loop. Such a mechanism could be involved in optimization of muscle development through induction of fusion processes (SIRT3) and preservation of a sufficient myoblast proliferation period (SIRT1). In addition, this result established that the inhibition of differentiation demonstrated in SIRT3 depleted myoblasts is not mediated through upregulation of SIRT1.

As SIRT3 deacetylates mitochondrial proteins and stimulates organelle activity [9], one interesting hypothesis would be that SIRT3 may affect myoblast differentiation through the control of mitochondrial activity and/or biogenesis. In agreement with other studies, our findings reveal that the mitochondrial activity increased from cell confluence to three days of differentiation, as reflected by significant increases in citrate synthase, complex II and cytochrome oxidase maximal activities, and maximal respiration, in control cells. This could result from the upregulation of the organelle biogenesis occurring during terminal differentiation. Indeed, we observed an increase in the expression of PGC-1α, a well-known regulator of mitochondriogenesis [36].

SIRT3 depletion significantly inhibited basal and maximal mitochondrial respiration, as well as citrate synthase, complex II and cytochrome oxidase maximal activities. This reduction of the organelle activity could thus be explained by the inhibition of mitochondrial biogenesis and/or the inability of SIRT3 to deacetylate several individual proteins inside mitochondria. In line with this hypothesis, the activity of complex II that comprises a subunit (SdhA) specifically deacetylated by SIRT3 [19], [37] is affected by SIRT3 depletion. Moreover, the expression of PGC-1α is decreased in SIRT3 depleted cells. A decrease in PGC-1α expression was previously reported in skeletal muscle of SIRT3-deficient mice [38] suggesting a potential regulation of mitochondrial biogenesis by SIRT3.

As well, we wanted as well to answer whether SIRT3 myogenic activity was essentially mediated through its control of mitochondrial function. Several results argued in favor of this hypothesis: i) through deacetylation defects, SIRT3 depletion probably inhibited the activity of specific proteins inside the organelle leading to a decreased mitochondrial activity [39]; ii) inhibition of mitochondrial protein synthesis induces a functional deficiency of the organelle and a differentiation arrest mediated by inhibition of Myogenin expression [2], [4]; iii) similarly, SIRT3 depletion significantly reduces Myogenin expression. However, while inhibition of mitochondrial protein synthesis did not affect MyoD protein expression [2], [4], SIRT3 depletion led to a decrease in the expression of the myogenic factor. Taken together, these results suggested that besides its direct influence on the organelle activity, SIRT3 could act *via* an additional mechanism. In agreement with Jing et al. [40], we report a rise in ROS production in SIRT3 depleted myoblasts related to the decrease of MnSOD activity. Given that MnSOD is a target of SIRT3 [16], [17], this result indicates a possible hyperacetylation of the antioxidant enzyme in depleted SIRT3 cells. ROS are not only detrimental products leading to alterations in lipids, proteins and DNA molecules, but also second messengers involved in cell signaling [41], they could therefore participate in this additional mechanism. Intracellular ROS affect the phosphorylation, activation, oxidation, and DNA-binding ability of various transcription factors including activator protein 1 (AP-1), nuclear factor kappa B (NF-κB), p53, leading to changes in their target gene expression [42]. In myoblasts, it has been shown that inducers of NF-κB (p65) reduced or blocked terminal differentiation by decreasing mRNA expression and stability of MyoD [43]–[46]. Thus, the increased ROS level observed in shSIRT3 myoblast mitochondria could result in the downregulation of the myogenic transcription factor MyoD expression through activation of NF-κB. The potential role of NF-κB as a downstream effector of shSIRT3-mediated skeletal muscle dysfunction should therefore be further explored.

In conclusion, this study reported the first results concerning the expression of SIRT3 during myoblast differentiation and established that this sirtuin is a potent positive regulator of the myogenic differentiation. This regulation resulted from its specific activity inside the mitochondria. In addition, we have demonstrated the occurrence of a crosstalk between SIRT3 and SIRT1, able to induce fine tuning of differentiation. Apart from its recognized metabolic role, SIRT3 could therefore be a crucial regulator of muscle differentiation.

## References

[1] Ljubicic V, Joseph A, Saleem A, Uguccioni G, Collu-Marchese M, et al (2010) Transcriptional and post-transcriptional regulation of mitochondrial biogenesis in skeletal muscle: Effects of exercise and aging. Biochem Biophys Acta 1800:223–234. 19682549 10.1016/j.bbagen.2009.07.031

[2] Rochard P, Rodier A, Casas F, Cassar-Malek I, Marchal-Victorion S, et al (2000) Mitochondrial activity is involved in the regulation of myoblast differentiation through myogenin expression and activity of myogenic factors. J Biol Chem 275:2733–2744. 10644737 10.1074/jbc.275.4.2733

[3] Duguez S, Sabido O, Freyssenet D (2004) Mitochondrial-dependent regulation of myoblast proliferation. Exp Cell Res 299:27–35. 15302570 10.1016/j.yexcr.2004.05.017

[4] Seyer P, Grandemange S, Busson M, Carazo A, Gamaleri F, et al (2006) Mitochondrial activity regulates myoblast differentiation by control of c-myc expression. J Cell Physiol 207:75–86. 16261590 10.1002/jcp.20539

[5] Seyer P, Grandemange S, Rochard P, Busson M, Pessemesse L, et al (2011) P43-dependant mitochondrial activity regulates myoblast differentiation and slow myosin isoform expression by control of calcineurin expression. Exp Cell Res 317:2059–2071. 21664352 10.1016/j.yexcr.2011.05.020

[6] Houtkooper RH, Pirinen E, Auwerx J (2012) Sirtuins as regulators of metabolism and healthspan. Nat Rev Mol Cell Biol 13:225–238. 22395773 10.1038/nrm3293 PMC4872805

[7] Fulco M, Schiltz RL, Lezzi S, King MT, Zhao P, et al (2003) Sir2 regulates skeletal muscle differentiation as a potential sensor of the redox state. Mol Cell 12:51–62. 12887892 10.1016/s1097-2765(03)00226-0

[8] Fulco M, Cen Y, Zhao P, Hoffman EP, McBurney MW, et al (2008) Glucose restriction inhibits skeletal myoblast differentiation by activating Sirt1 through AMPK-mediated regulation of Nampt. Dev Cell 14:661–673. 18477450 10.1016/j.devcel.2008.02.004 PMC2431467

[9] Verdin E, Hirschey MD, Finley LWS, Haigis MC (2010) Sirtuin regulation of mitochondria: energy production, apoptosis, and signaling. Trends Biochem Sci 35:669–675. 20863707 10.1016/j.tibs.2010.07.003 PMC2992946

[10] Lombard DB, Alt FW, Cheng HL, Bunkenborg J, Streeper RS, et al (2007) Mammalian Sir2 homolog SIRT3 regulates global mitochondrial lysine acetylation. Mol. Cell Biol 27:8807–8814. 10.1128/MCB.01636-07 PMC2169418 17923681

[11] Haigis MC, Mostoslavsky R, Haigis KM, Fahie K, Christodoulou DC, et al (2006) SIRT4 inhibits glutamate dehydrogenase and opposes the effects of calorie restriction in pancreatic beta cells. Cell 126:941–54. 16959573 10.1016/j.cell.2006.06.057

[12] Du J, Zhou Y, Su X, Yu JJ, Khan S, et al (2011) Sirt5 is a NAD-dependent protein lysine demalonylase and desuccinylase. Science 334:806–809. 22076378 10.1126/science.1207861 PMC3217313

[13] Hallows WC, Lee S, Denu JM (2006) Sirtuins deacetylase and activate mammalian acetyl-CoA synthetases. Proc Natl Acad Sci 103:10230–10235. 16790548 10.1073/pnas.0604392103 PMC1480596

[14] Schwer B, Bunkenborg J, Verdin RO, Andersen JS, Verdin E (2006) Reversible lysine acetylation controls the activity of the mitochondrial enzyme acetyl-coA synthetase 2. Proc Natl Acad Sci 103:10224–10229. 16788062 10.1073/pnas.0603968103 PMC1502439

[15] Hirschey MD, Shimazu T, Goetzman E, Jing E, Schwer B, et al (2010) SIRT3 regulates mitochondrial fatty-acid oxidation by reversible enzyme deacetylation. Nature 464:121–125. 20203611 10.1038/nature08778 PMC2841477

[16] Qiu X, Brown K, Hirschey MD, Verdin E, Chen D (2010) Calorie restriction reduces oxidative stress by SIRT3-mediated SOD2 activation. Cell Metabolism 12:662–667. 21109198 10.1016/j.cmet.2010.11.015

[17] Tao R, Coelman MC, Pennington D, Ozden O, Park S, et al (2010) Sirt3-Mediated Deacetylation of Evolutionarily Conserved Lysine 122 Regulates MnSOD Activity in Response to Stress. Mol Cell 40:893–904. 21172655 10.1016/j.molcel.2010.12.013 PMC3266626

[18] Ahn BH, Kim HS, Song S, Lee IH, Liu J, et al (2008) A role for the mitochondrial deacetylase sirt3 in regulating energy homeostasis. Proc Natl Acad Sci 105:14447–14452. 18794531 10.1073/pnas.0803790105 PMC2567183

[19] Cimen H, Han MJ, Yang Y, Tong Q, Koc H, et al (2010) Regulation of succinate dehydrogenase activity by SIRT3 in mammalian mitochondria. Biochemistry 49:304–311. 20000467 10.1021/bi901627u PMC2826167

[20] Van de Wetering M, Oving I, Muncan V, Pon Fong MT, Brantjs H, et al (2003) Specific inhibition of gene expression using a stably integrated, inducible small-interfering-RNA vector. EMBO Rep 4:609–615. 12776180 10.1038/sj.embor.embor865 PMC1319205

[21] Davis RL, Weintraub H, Lassar AB (1987) Expression of a single transfected cDNA converts fibroblasts to myoblasts. Cell 51:987–1000. 3690668 10.1016/0092-8674(87)90585-x

[22] Frezza C, Cipolat S, Scorrano L (2007) Organelle isolation: functional mitochondria from mouse liver, muscle and cultured filroblasts. Nat Protoc 2:287–295. 17406588 10.1038/nprot.2006.478

[23] Rustin P, Chretien D, Bourgeron T, Gerard B, Rotig A, et al (1994) Biochemical and molecular investigations in respiratory chain deficiencies. Clin Chim Acta 228:35–51. 7955428 10.1016/0009-8981(94)90055-8

[24] Wharton DC, Tzagoloff A, Ronald W, Estabrook MEP (1967) Cytochrome oxidase from beef heart mitochondria. Methods in Enzymology Academic Press 45:245–250.

[25] Srere P (1969) Citrate synthase: [EC 4.1.3.7]. Citrate oxaloacetate-lyase (Co acetylating). Methods in Enzymology Academic Press 1:3–11.

[26] Marklund S (1976) spectrophotometric study of spontaneous disproportionation of superoxide anion radical and sensitive direct assay for superoxide dismutase. J Biol Chem 251:7504–7507. 12168

[27] Gnaiger E (2001) Bioenergetics at low oxygen: dependence of respiration and phosphorylation on oxygen and adenosine diphosphate supply. Respir Physiol 128:277–297. 11718759 10.1016/s0034-5687(01)00307-3

[28] Laguerre M, Wrutniak-Cabello C, Chabi B, López Giraldo LJ, Lecomte J, et al (2011) Does hydrophobicity always enhance antioxidant drugs? A cut-off effect of the chain length of functionalized chlorogenate esters on ROS-overexpressing fibroblasts. J Pharm Pharmacol 63:531–40. 21401605 10.1111/j.2042-7158.2010.01216.x

[29] Nemoto S, Fergusson MM, Finkel T (2005) SIRT1 functionally interacts with the metabolic regulator and transcriptional coactivator PGC-1 {alpha}. J Biol Chem 280:164456–164460. 10.1074/jbc.M501485200 15716268

[30] Perry RL, Rudnicki MA (2000) Molecular mechanisms regulating myogenic determination and differentiation. Front Biosci 5:D750–D767. 10966875 10.2741/perry

[31] Remels AH, Langen RC, Schrauwen P, Schaart G, Schols AM, et al (2010) Regulation of mitochondrial biogenesis during myogenesis. Mol Cell Endocrinol 315:113–120. 19804813 10.1016/j.mce.2009.09.029

[32] Scarpulla RC (2011) Metabolic control of mitochondrial biogenesis through the PGC-1 family regulatory network. Biochim Biophys Acta 1813:1269–1278. 20933024 10.1016/j.bbamcr.2010.09.019 PMC3035754

[33] Kraft CS, LeMoine CM, Lyons CN, Michaud D, Mueller CR, et al (2006) Control of mitochondrial biogenesis during myogenesis. Am J Physiol Cell Physiol 290:C1119–C1127. 16531567 10.1152/ajpcell.00463.2005

[34] Barbieri E, Battistelli M, Casadei L, Vallorani L, Picolli G, et al. (2011) Morphofunctional and biochemical approaches for studying mitochondrial changes during myoblasts differentiation. J Aging Res 2011: ID845379, 16 pages, doi:10.4061/2011/845379. 10.4061/2011/845379 PMC3100678 21629710

[35] Berkes CA, Tapscott SJ (2005) MyoD and the transcriptional control of myogenesis. Semin. Cell Dev Biol 16:585–595. 10.1016/j.semcdb.2005.07.006 16099183

[36] Wu Z, Puigserver P, Andersson U, Zhang C, Adelmant G, et al (1999) Mechanisms controlling mitochondrial biogenesis and respiration through the thermogenic coactivator PGC-1. Cell 98:115–124. 10412986 10.1016/S0092-8674(00)80611-X

[37] Finley LW, Haas W, Desquiret-Dumas V, Wallace DC, Procaccio V, et al (2011) Succinate dehydrogenase is a direct target of sirtuin 3 deacetylase activity. PLoS One 6:e23295. 21858060 10.1371/journal.pone.0023295 PMC3157345

[38] Palacios OM, Carmona JJ, Michan S, Chen KY, Manabe Y, et al (2009) Diet and exercise signals regulate SIRT3 and activate AMPK and PGC-1alpha in skeletal muscle. Aging 1:771–783. 20157566 10.18632/aging.100075 PMC2815736

[39] Zhong L, Mostoslavsky R (2011) Fine tuning our cellular factories: sirtuins in mitochondrial biology. Cell Metab 13:621–626. 21641544 10.1016/j.cmet.2011.05.004 PMC3111451

[40] Jing E, Emanuelli B, Hirschey MD, Boucher J, Lee KY, et al (2011) Sirtuin-3 (Sirt3) regulates skeletal muscle metabolism and insulin signaling via altered mitochondrial oxidation and reactive oxygen species production. Proc Natl Acad Sci 108:14608–14613. 21873205 10.1073/pnas.1111308108 PMC3167496

[41] Brookes PS, Yoon Y, Robotham JL, Anders MW, Sheu SS (2004) Calcium, ATP, and ROS: a mitochondrial love-hate triangle. Am J Physiol Cell Physiol 287:C817–C833. 15355853 10.1152/ajpcell.00139.2004

[42] Verschoor ML, Wilson LA, Singh G (2010) Mechanisms associated with mitochondrial generated reactive oxygen species in cancer Can J Physiol Pharmaco. 88:204–219. 10.1139/Y09-135 20393586

[43] Guttridge DC, Mayo MW, Madrid LV, Wang CY, Baldwin AS Jr (2000) NF-kB-induced loss of MyoD messenger RNA: possible role in muscle decay and cachexia. Science 289:2363–2366. 11009425 10.1126/science.289.5488.2363

[44] Langen RC, Schols AM, Kelders MC, Wouters EF, Janssen-Heininger YM (2001) Inflammatory cytokines inhibit myogenic differentiation through activation of nuclear factor-B. FASEB J 15:1169–1180. 11344085 10.1096/fj.00-0463

[45] Dogra C, Changotra H, Mohan S, Kumar A (2006) Tumor necrosis factor-like weak inducer of apoptosis inhibits skeletal myogenesis through sustained activation of nuclear factor-kB and degradation of MyoD protein. J Biol Chem 281:10327–10336. 16461349 10.1074/jbc.M511131200

[46] Wang H, Hertlein E, Bakkar N, Sun H, Acharyya S, et al (2007) NF-kB regulation of YY1 inhibits skeletal myogenesis through transcriptional silencing of myofibrillar genes. Mol Cell Biol 27:4374–4387. 17438126 10.1128/MCB.02020-06 PMC1900043

